# Defense–competition trade‐offs shape prey eco‐evolutionary dynamics across environmental gradients

**DOI:** 10.1002/ecy.70518

**Published:** 2026-09-23

**Authors:** Rafael Dettogni Guariento, Frederico Meirelles‐Pereira, Anderson da Rocha Gripp, Jayme Magalhães Santangelo, Luciana Silva Carneiro, Adriano Caliman

**Affiliations:** ^1^ Universidade Federal de Mato Grosso do Sul Campo Grande Mato Grosso do Sul Brazil; ^2^ Universidade Federal do Rio de Janeiro Rio de Janeiro Rio de Janeiro Brazil; ^3^ Universidade Federal Rural do Rio de Janeiro Seropédica Rio de Janeiro Brazil; ^4^ Universidade Federal do Rio Grande do Norte Natal Rio Grande do Norte Brazil

**Keywords:** adaptive dynamics, convergence stability, evolutionary stability, predation, prey competitiveness, prey defense

## Abstract

Predators can affect ecological communities not only through direct consumption but also by indirectly triggering costly defensive responses in prey that cascade through food webs. Understanding how prey navigate the competing demands of predation pressure and resource exploitation is thus central to predicting ecological (i.e., persistence) and evolutionary (i.e., adaptation) dynamics. Drawing on niche theory and the influence of keystone predators, this study investigates how variation in resource availability drives the persistence and evolution of prey with diverse trait combinations balancing defensive and competitive traits. At the heart of this ecological puzzle lies a fundamental constraint: Defended prey inherently have lower competitive abilities, generating a trade‐off that governs fitness success along environmental gradients. Using a mathematical model, we show that the shape of the defense–competitiveness trade‐off (i.e., the magnitude of change of competitiveness costs with increasing defense) influences the persistence of prey populations and the evolution of prey competitive and defensive traits. Strong trade‐offs (i.e., when defenses strongly reduce competitiveness) or neutral trade‐offs (i.e., when gains in defense and losses in competitiveness change proportionally) favor competitiveness, while weak trade‐offs (i.e., when defenses weakly affect competitiveness) favor defensive traits, especially in resource‐rich environments. In resource‐poor environments, however, competitive prey are predominantly favored. Coexistence among prey individuals with different resource allocation strategies is evolutionarily unstable, as our model revealed that evolutionary processes eroded the persistence of multiple prey strategies. By linking resource availability to prey trait evolution, this study highlights the environmental dependence of eco‐evolutionary dynamics in food webs, offering insights into the complex interactions that drive the prevalence and evolution of defense strategies.

## INTRODUCTION

Ecological models based on traits, characteristics describing the morphology, biochemistry, physiology, phenology, or behavior of an organism (Violle et al., 2007), have advanced our ability to explain and predict community structure and dynamics across environmental gradients (Litchman et al., 2007). Such trait‐based frameworks are particularly valuable for understanding predator–prey interactions. These interactions are influenced by covarying traits among interacting organisms and the trade‐offs they entail. There is growing evidence that correlations among prey traits, especially between defense and competitive abilities, play a key role in determining trophic outcomes in food webs (Réveillon & Becks, 2024).

Prey commonly show context‐dependent trait adaptation, adjusting morphological or behavioral traits to maximize fitness across combinations of resource availability and predation pressure (Wahlström et al., 2000). The expression of defensive traits often incurs costs, modifications in behavior, life history, morphology, or physiology that can reduce competitive fitness through decreased resource acquisition, resource use efficiency, and/or reproduction (Halsey & Jones, 2015). The competitive costs of defense do not always translate into net fitness costs, but they can impose selective pressures that often exceed the impact of direct predation (Creel & Christianson, 2008). Consequently, understanding the balance between these constraints and their fitness costs is key to unraveling optimal fitness strategies and understanding the mechanisms that generate and maintain intraspecific variation within prey populations.

The defense–competitiveness trade‐off may be linear, where greater defense investment yields proportional reductions in competitive abilities (Réveillon & Becks, 2024). However, defensive responses often show nonlinear patterns. For example, the marginal costs or benefits of additional defense may decline as investment increases, as in vigilance behavior (Pulliam, 1973), and grouping patterns (Caro, 2005). These different cost–benefit shapes mean that the payoff of investing in defenses varies with environmental conditions, sometimes favoring competitive ability over defenses, and other times the reverse (Preisser & Bolnick, 2008). Understanding how trade‐off relationships in prey defense and competitive traits vary across different environmental conditions is essential to unraveling intraspecific trait variation, which can be equally important for ecological dynamics as interspecific variation (Des Roches et al., 2018).

Contemporary niche theory offers a framework to elucidate how environmental factors modulate the expression of traits related to competitive ability and to defense (Chase & Leibold, 2003). Based on resource competition theory (Tilman, 1980), this framework recognizes that biophysicochemical factors indirectly influence species interactions through environmental feedbacks. Moreover, niche theory offers insight into the eco‐evolutionary mechanisms driving intraspecific dynamics and trait evolution across gradients (Koffel et al., 2016). Studies have shown that the shape of trait trade‐offs can determine species coexistence and community dynamics by altering how traits translate into fitness (e.g., Ehrlich et al., 2017). For example, Ehrlich et al. (2020) showed how a weak defense‐growth trade‐off in phytoplankton governs seasonal trait dynamics, favoring species with intermediate defense under certain conditions. However, there is limited empirical and theoretical work exploring how the shape of the defense–competition trade‐off interacts with resource supply to determine the evolution and stability of prey traits. Unlike studies analyzing resource allocation between defense types in plants, such as tolerance versus resistance (Ehrlich et al., 2017, 2020; Koffel et al., 2018), the interplay between resource availability and these defense–competitiveness trade‐offs in animal prey may follow distinct dynamics. Whereas plants counter herbivory through various mechanisms, including enhancing their growth/reproduction to numerically counteract the losses by herbivory (i.e., tolerance) (Monson et al., 2022) or defense–competitiveness trade‐offs, animals typically evolve behavioral or morphological defenses that may impose opportunity costs (i.e., losses of alternative reproductive or foraging options) on their ability to acquire resources (Preisser & Bolnick, 2008).

In this study, we investigate how prey strategies, characterized by the balance between competitiveness for resources and defense against predators, vary along resource gradients using a mathematical and graphical framework based on a three‐level diamond food web (Koffel et al., 2016; Leibold, 1996; Tilman, 1980), comprising predators, an animal herbivore, and primary producers. Using an eco‐evolutionary model, we examine how resource availability and the shape of the prey defense–competition trade‐off jointly shape selection on prey defense, determining the evolutionarily singular defense level (i.e., the trait value at which the selection gradient vanishes) and whether that level is convergence stable (i.e., defense singular level converges evolutionarily) and evolutionarily stable (i.e., resists invasion by mutants). We also evaluate how these properties are reflected in the prevalence of alternative prey strategies. We first characterize the prey defense–competition trade‐off across alternative allocations to defense versus competitive traits, distinguishing effects on direct predation from the costs on prey foraging. We then examine how these traits generate environmental feedbacks. Specifically, we test how environmental conditions shape trait values and trade‐offs by evaluating population persistence across resource availability levels. This clarifies the selective pressures shaping prey trait allocation in an eco‐evolutionary framework, and we analyze how such feedback influences their evolution. Our study offers two complementary approaches on the dynamics of prey competitiveness and defense. The first focuses on trait sorting and intraspecific interactions within a finite trait space, providing an abstract framework for understanding the persistence of traits in heterogeneous populations. The second examines invasion dynamics in a local neighborhood of prey with distinct traits surrounding a resident prey population, highlighting conditions that may give rise to stable prey trait allocation strategies.

## METHODS

### Model structure

We developed a model grounded in contemporary niche theory to describe prey populations in a dynamic population–environment system (Chase & Leibold, 2003). The mechanistic equivalence between species sorting in communities and clonal sorting in asexual populations (Klauschies et al., 2016) means that models developed for interspecific competition and community dynamics can be directly used to describe trait dynamics within a single population. Using this logic, we adapt the graphical and analytical tools of contemporary niche theory, originally designed for species coexistence along environmental gradients, to examine sorting and evolution of prey phenotypes within a population. Here, prey “populations” represent individuals with different trait allocations (analogous to clones or genotypes within one species), and their environment consists of a primary producer (resource) and a top predator (consumer).

Our model builds on the diamond‐shaped food web framework (Koffel et al., 2018; Leibold, 1996), extending it to focus on distinct prey phenotypes at an intermediate trophic level. Unlike previous models centered on phytoplankton (Ehrlich et al., 2020; Koffel et al., 2018), our framework incorporates consumers feeding on logistically growing resources, capturing the dual challenge of resource acquisition and predator avoidance faced by herbivores or mesopredators (Guariento et al., 2022). It also refines the understanding of predator–prey interactions by explicitly parameterizing the trade‐off between prey defense and resource acquisition (Creel & Christianson, 2008; Ehrlich et al., 2017) and by incorporating environmental context to shape the trade‐off strategies. To capture both ecological and evolutionary perspectives, we employ two analytical approaches. The ecological approach investigates which trait combinations persist under different resource supplies by evaluating competition among fixed strategies. The evolutionary approach explores adaptive changes in trait values via invasion analysis, identifying which strategies produce stable dynamics and are evolutionarily selected. Together, these methods clarify the conditions selecting for different prey phenotypes and their long‐term persistence across environmental gradients.

Our three‐level diamond food web represents a collection of n distinct prey trait variants $N_{j}$ (j=1,…,n), consuming a resource R and being consumed by a single predator species P. We model the dynamics using the following ordinary differential equations:

(1a) $$\frac{\mathrm{dR}}{\mathrm{dt}}=\left(r-\lambda R\right)R-\sum_{j=1}^{n}g_{j}N_{j}R,$$

(1b) $$\frac{{\mathrm{dN}}_{j}}{\mathrm{dt}}=e_{N}g_{j}{\mathrm{RN}}_{j}-f_{j}{\mathrm{PN}}_{j}-d_{N}N_{j},$$

(1c) $$\frac{\mathrm{dP}}{\mathrm{dt}}=-d_{P}P+\sum_{j=1}^{n}e_{P}f_{j}N_{j}P.$$

The system is closed (i.e., no immigration or emigration). All state variables and parameters, as well as their numerical values and units, are summarized in Appendix S1: Table S1. Equation (1a) describes resource dynamics. The resource grows logistically with intrinsic rate r and intraspecific competition coefficient λ (Pianka, 1972).

Equation (1b) gives prey dynamics. Prey gain biomass at a per capita rate $e_{N}g_{j}R$, where $g_{j}$ captures resource acquisition and $e_{N}$ is the conversion efficiency (i.e., the proportion of resource ingested that is assimilated and used for maintenance, growth, and reproduction) from ingested resource to prey growth (Litchman et al., 2007). Losses arise from two terms: predation through a mass‐dependent attack rate with attack rate $f_{j}$ (Guariento et al., 2022), and an inherent, predator‐independent mortality rate $d_{N}$.

Equation (1c) tracks predator dynamics. Predators increase through consumption of prey at attack rate $f_{j}$, converted to predator biomass with efficiency $e_{P}$, and decline at a constant mortality rate $d_{P}$. The functional‐response assumptions used here align with previous work on modern coexistence theory (Chesson & Kuang, 2008) and capture realistic scenarios for filter feeders (Jeschke et al., 2004), including fish/crustacean interactions (Novak et al., 2025). The model was parameterized on an algae–zooplankton–fish system (Appendix S1: Table S1).

Because the growth function for resources is nonlinear (i.e., logistic growth), it does not fit the general framework of the classical graphical approach implemented for contemporary niche theory (Koffel et al., 2021). Yet, the theory can be directly adapted by factoring the problem as follows:

(2) $$\frac{\mathrm{dR}}{\mathrm{dt}}=\lambda \left(K-R\right)R-\sum_{j=1}^{n}g_{j}N_{j}R,$$

with $K=\frac{r}{\lambda }$, representing the carrying capacity of the resource population. For an accurate visualization in the graphical approach (see below), the impact vector must be normalized to account for the new turnover rates (Koffel et al., 2021).

### The ecological approach

Here, we use the ecological approach to ask which prey strategies can persist across the resource gradient when trait values are fixed. This analysis establishes a feasibility baseline by isolating how bottom‐up resource supply and top‐down predation jointly constrain prey persistence before allowing traits to evolve. To evaluate strategy prevalence under fixed (non‐evolving) traits, we graphically identify ecological equilibria along resource supply gradients, following Tilman (1980) and Leibold (1996). Our approach involves two steps: (1) invasion analysis, which determines equilibrium conditions allowing prey with distinct traits to grow and persist, and (2) supply point mapping, which locates points along gradients where these conditions are met (Koffel et al., 2016). This analysis centers on three elements: supply points, zero net growth isoclines (ZNGIs), and impact vectors.

We determined the ZNGIs and supply points to analyze the equilibrium of the system. For the graphical construction of these equilibria, please refer to Appendix S2, and for detailed mathematical derivations and stability analysis, see Appendix S3. This graphical approach proves especially valuable when analyzing distinct prey phenotypes along the resource gradient (Appendix S2: Figure S1). Multi‐prey invasion analysis overlays their ZNGIs: Coexistence between two prey phenotypes occurs only at ZNGI intersections, requiring a trade‐off where one prey shows superior competitive ability (i.e., lower $R^{*}$), while the other demonstrates greater defense against predators (i.e., steeper ZNGI slope). This trade‐off between defense and competitiveness can emerge from trait allocation models, as shown in the “Trait Allocation Strategy” section below. Combining uninvasible ZNGI portions with their impact vectors produces a multi‐prey bifurcation diagram that delineates regions of single‐phenotype persistence and coexistence along the resource gradient (Appendix S2: Figure S1B).

### Trait allocation strategy

To capture how the change in one trait value changes the other trait value and how it drives prey growth, we introduce the following conversion function for the prey population *per capita* growth rate:

(3) $$w\left(x,R,P\right)=e_{N}\;g\;x\;R-f\;x^{\alpha }\;P-d_{N}.$$

**FIGURE 1:**
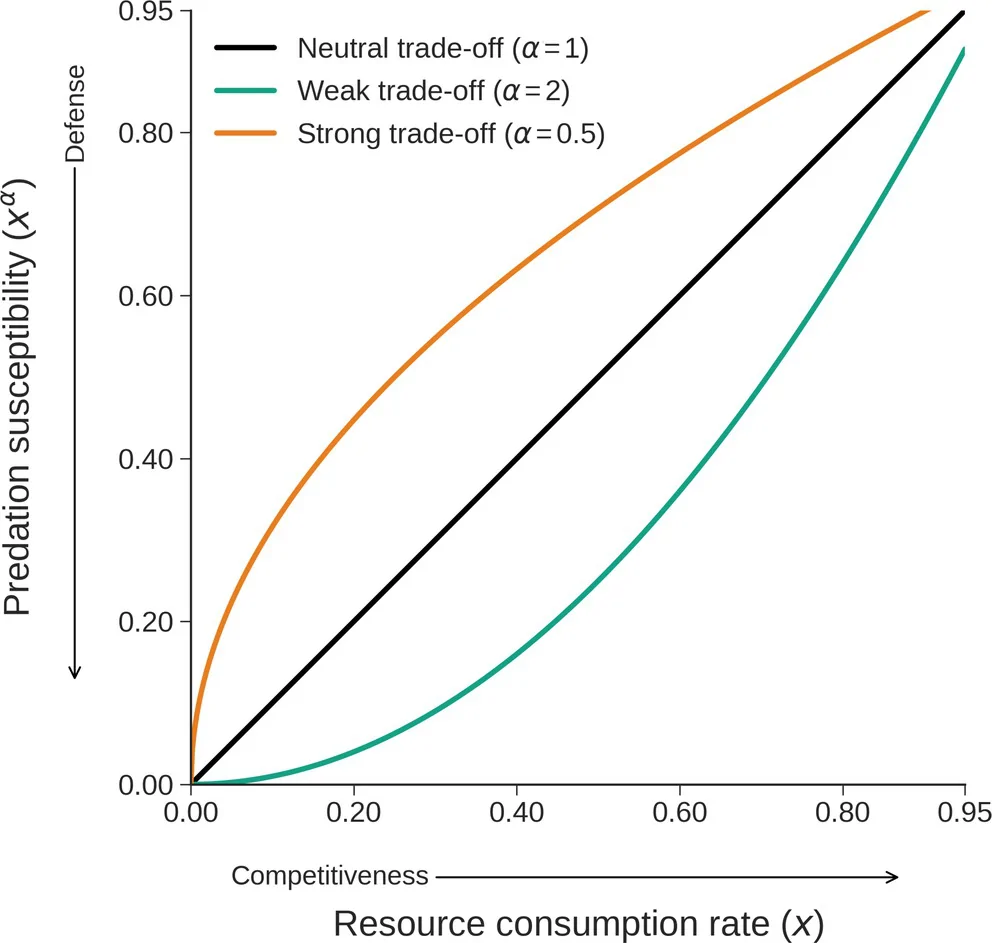
Defense–competitiveness trade‐off for various shape parameters α. This relationship is linear when α=1 (neutral trade‐off), concave when α<1 (strong trade‐off), and convex when α>1 (weak trade‐off).

In this equation, we introduce two parameters: the prey trait allocation strategy (x) and the trade‐off shape parameter (α). The parameter x, which ranges from 0 to 1, determines how the prey allocate resources between defense and competitiveness. As x increases, the prey consumption rate approaches its maximum value, but the predator attack rate on the prey also increases toward its maximum value. Therefore, higher values of x make the prey more resource‐competitive but more vulnerable to predation, while lower values of x make the prey less resource‐competitive but less vulnerable to predation. In both cases, the benefits of one aspect lead to the compromise of the other. In this formulation, the trait allocation strategy x maps to the rates in system (1) as $g_{j}=\mathrm{gx}$ and $f_{j}={\mathrm{fx}}^{\alpha }$. However, the magnitude of x and the predator maximum attack rate on prey can scale nonlinearly, depending on the shape parameter α, which governs the shape of the trade‐off. The trade‐off curve defines the boundary of the feasible trait combination set, encompassing all potential prey phenotypes (Ehrlich et al., 2020). This curve is constrained by individual‐level limitations, such as physiological restrictions, and can take various forms (e.g., concave or convex, Figure 1), reflecting differing costs of trait adjustments (Rueffler et al., 2006). In zooplankton, a two‐dimensional trait space can represent both defense against predators and resource acquisition (i.e., competitiveness), an essential trade‐off faced by prey individuals (Kiørboe, 2011). When α=1, predation susceptibility increases linearly with resource acquisition. This also represents the case where defense decreases linearly with resource acquisition. When α>1, increasing resource acquisition produces a less‐than‐proportional increase in predation susceptibility, so the predation cost rises relatively slowly at first. When α<1, increasing resource acquisition produces a greater‐than‐proportional increase in predation susceptibility, so the predation cost rises relatively quickly at first (Figure 1). We distinguish three cases of defense–competitiveness trade‐offs, inspired by previous work (Creel & Christianson, 2008; Rueffler et al., 2004): (1) Neutral if the trade‐off is linear (i.e., α=1); (2) Weak if the trade‐off is convex (i.e., α>1); (3) Strong if the trade‐off is concave (i.e., α<1). This approach is crucial in understanding how selective pressures change along such gradients and ultimately drive prey adaptation. Traditionally, convex trade‐offs are referred to as strong, while concave trade‐offs are considered weak (Rueffler et al., 2004). However, our definition differs because in our representation of the trade‐off curve, the *y*‐axis represents the effects of predation on the growth of the prey population rather than defense (Figure 1). Consequently, fitness decreases as the *y*‐axis value increases.

### Eco‐evolutionary approach

Building on the feasibility baseline defined by the ecological analysis, the eco‐evolutionary approach examines which viable strategies are favored when trait values can evolve. Here, persistence is ecologically determined, while invasion analysis identifies long‐term evolutionary end points and their stability by examining dynamics within the local neighborhood of strategies surrounding a resident population. Rather than evaluating competition among prey with fixed traits, this approach examines how small, heritable mutations shift trait values toward evolutionarily stable strategies. Following the adaptive dynamics framework (Geritz et al., 1997, 1998), the invasion fitness w of an invading strategy with traits $x^{\prime }$ in a resident environment $\hat{E}$ ($\hat{R},\hat{P}$) is given by

(4) $$w\left(x^{\prime },\hat{E}\right)=\frac{1}{N}\frac{\mathrm{dN}}{\mathrm{dt}}=e_{N}\;g\;x^{\prime }\hat{R}-f\;{x^{\prime }}^{\alpha }\;\hat{P}-d_{N},$$

where the environment $\hat{E}$ is implicitly determined by the ecological equilibrium of the resident strategy x. This feedback loop between the evolving population and its environment leads to density‐ and frequency‐dependent selection (Koffel et al., 2018).

Introducing the fitness gradient:

(5) $${\left.s^{\prime }\left(x\right)=\frac{\partial w}{\partial x^{\prime }}\right|}_{x^{\prime }=x}.$$

Evolutionary equilibria, or singular strategies $\hat{x}$, are found by solving $s^{\prime }\left(x\right)=0$, where the selection gradient vanishes (Geritz et al., 1997). Stability is assessed locally through second‐order derivatives of invasion fitness w, and globally by evaluating w across trait space (Geritz et al., 1998). A trait allocation strategy x is a (local) evolutionarily stable strategy (ESS) if the second derivative of the fitness function at x is negative. ESSs are key because they cannot be invaded by alternative strategies. When eco‐evolutionary dynamics converge toward an ESS, it becomes a convergence stable strategy (CSS), representing a stable evolutionary equilibrium. Following Geritz et al. (1998), we used a graphical approach to identify singular strategies and assess their stability via pairwise invasibility plots (PIPs). A summary of the correspondence between singular strategy classifications and their graphical representations in the PIP is provided in Appendix S4.

Finally, the geometrical envelope of the ZNGI family (Koffel et al., 2016) generalizes graphical invasion analysis to a continuous distribution of distinct competing prey phenotypes, by treating the envelope as the boundary traced by the complete set of individual ZNGIs and using its geometry to characterize local invasibility. The mathematical derivation and graphical representation of this envelope are presented in Appendix S5.

## RESULTS

### Ecological analysis

**FIGURE 2:**
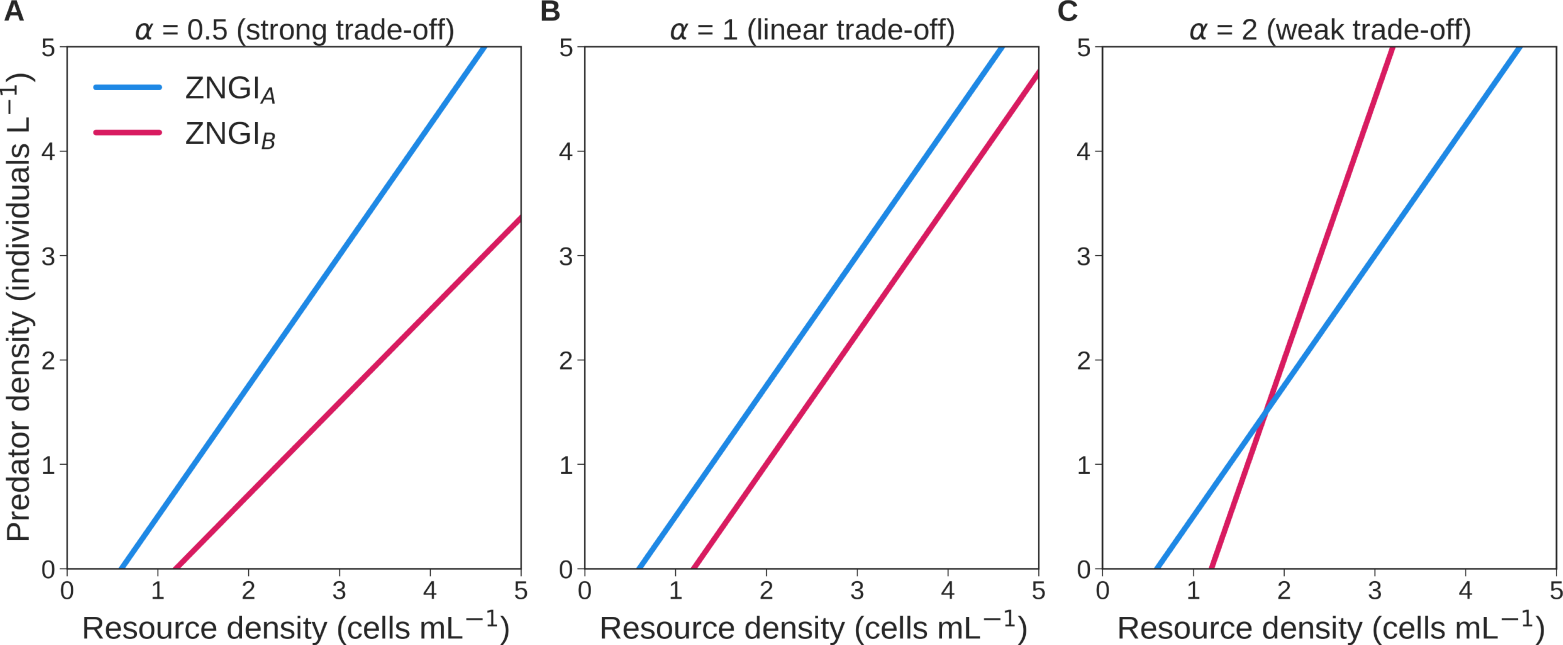
Zero net growth isoclines (ZNGIs) for two prey phenotypes exhibiting distinct survival strategies. The blue phenotype is more competitive and less defended, while the magenta phenotype is less competitive and more defended. Remaining parameters are the same as in Appendix S1: Table S1. The ZNGIs are shown under three scenarios of the defense–competitiveness trade‐off (α): (A) strong trade‐off, where specialization in either strategy carries significant costs; (B) neutral trade‐off, representing balanced costs between strategies; and (C) weak trade‐off, where populations can maintain relatively high performance in both strategies. Each ZNGI marks the boundary where the per capita growth rate of the corresponding prey equals zero (${\mathrm{dN}}_{j}/\mathrm{dt}=0$); for combinations of resource and predator densities below its own ZNGI a prey population/phenotype experiences positive growth $\left({\mathrm{dN}}_{j}/\mathrm{dt}>0\right)$, and above it, negative growth (${\mathrm{dN}}_{j}/\mathrm{dt}<0$). However, when two ZNGIs are plotted together, the shared equilibrium $\left(\hat{R},\hat{P}\right)$ lies on the outermost ZNGI, so the prey with the lower ZNGI experiences negative growth throughout the plotted region (${\mathrm{dN}}_{j}/\mathrm{dt}<0$), including regions where it would grow positively if considered alone.

**FIGURE 3:**
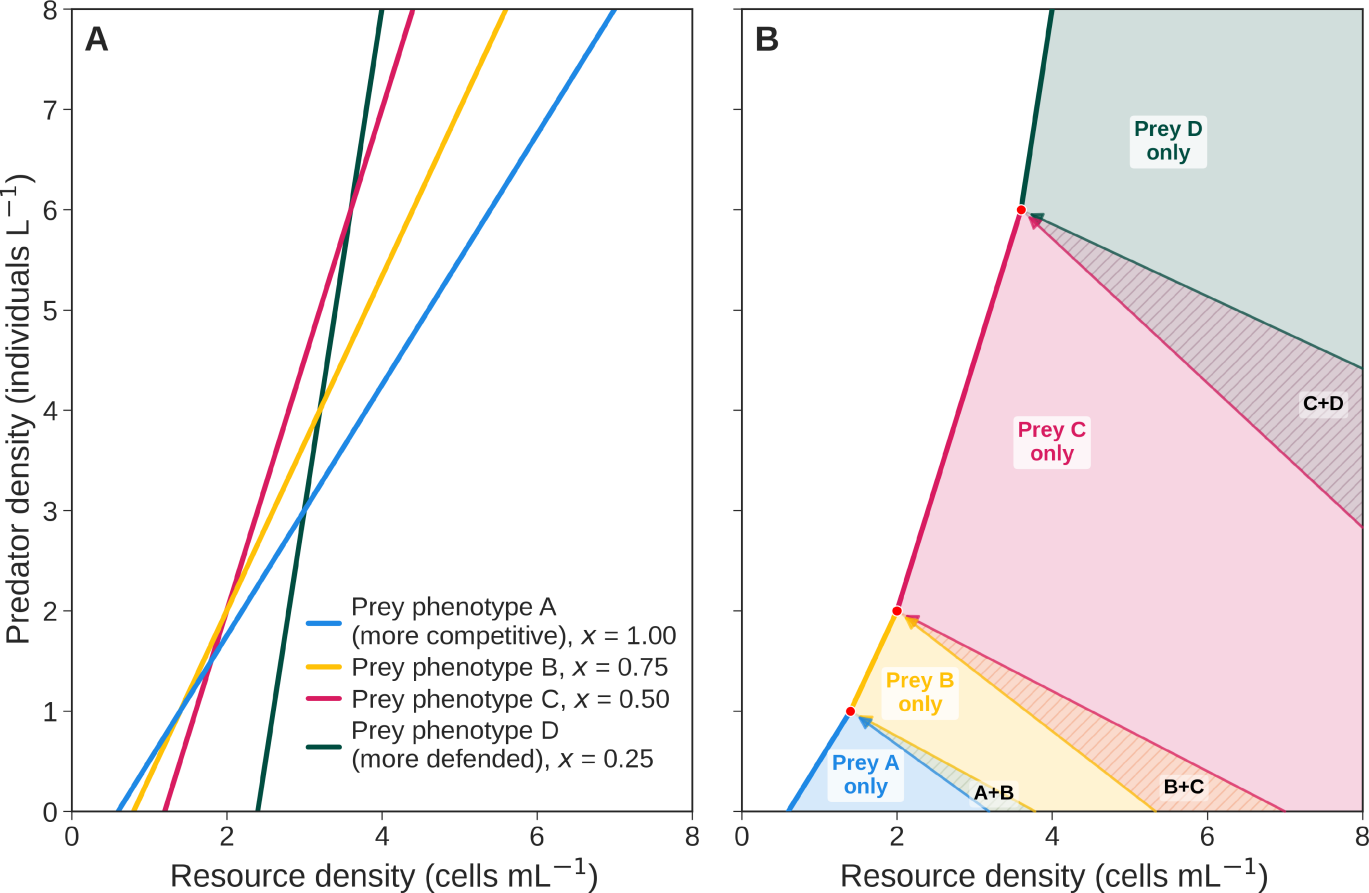
Ecological bifurcation diagrams for four prey phenotypes (A–D) under weak trade‐off conditions (α>1). The trait x ranges from 0 to 1 and defines the allocation strategy between defense and competitiveness. (A) Zero net growth isoclines (ZNGIs) for distinct defense–competitiveness trait combinations. Each ZNGI represents the combinations of resource and predator supply for which the growth rate of a prey phenotype is zero. All unspecified parameters are given in Appendix S1: Table S1. (B) Composite ZNGI diagram constructed from the outermost segments of the ZNGIs in Panel A, highlighting three critical intersection points (red dots) where multiple ZNGIs meet and potential coexistence equilibria may occur. Thin arrows indicate impact vectors originating from resource supply conditions and delimit the regions in which different prey–phenotype pairs (A+B, B+C, or C+D) can stably coexist. The boundaries of these regions are determined by the relative strengths of resource competition and defense among the constituent phenotypes.

The ecological results describe how prey trait combinations are sorted along the resource gradient when trait values are fixed, identifying which strategies can persist under different combinations of resource supply and predator abundance. When analyzing the interaction of two prey phenotypes with different resource allocation strategies, constrained by the defense–competitiveness trade‐off, we identified unique patterns (Figure 2). For strong trade‐offs (α=0.5) and neutral trade‐offs (α=1), the strategy that maximizes resource uptake (blue lines, Figure 2A,B) was always favored. This is because, in the proposed model formulation, the ZNGIs never intersect for positive values of *R* and *P* when α≤1, regardless of remaining parameter values. Conversely, weak trade‐offs (α=2) uniquely allow ZNGIs to intersect (Figure 2C), enabling coexistence of two prey phenotypes with distinct strategies. To explore the generality of this coexistence result and examine how a broader range of trait allocations are sorted along the resource gradient, we extended the analysis to four prey phenotypes spanning a wider portion of the defense–competitiveness trait space (Figure 3A,B). This extension reveals not only pairwise coexistence but also how the identity of the dominant strategy shifts with resource availability. Importantly, coexistence under weak trade‐offs occurs only among prey with relatively similar trait allocations (Figure 3B). Numerical simulations corroborate this pattern: Appendix S2: Figure S2 shows seven supply points that span distinct community composition regions in the ZNGI map, and Appendix S2: Figure S2 shows the corresponding equilibria shifting from competitively biased to more defended strategies as resource supply increases.

### Eco‐evolutionary analysis

**FIGURE 4:**
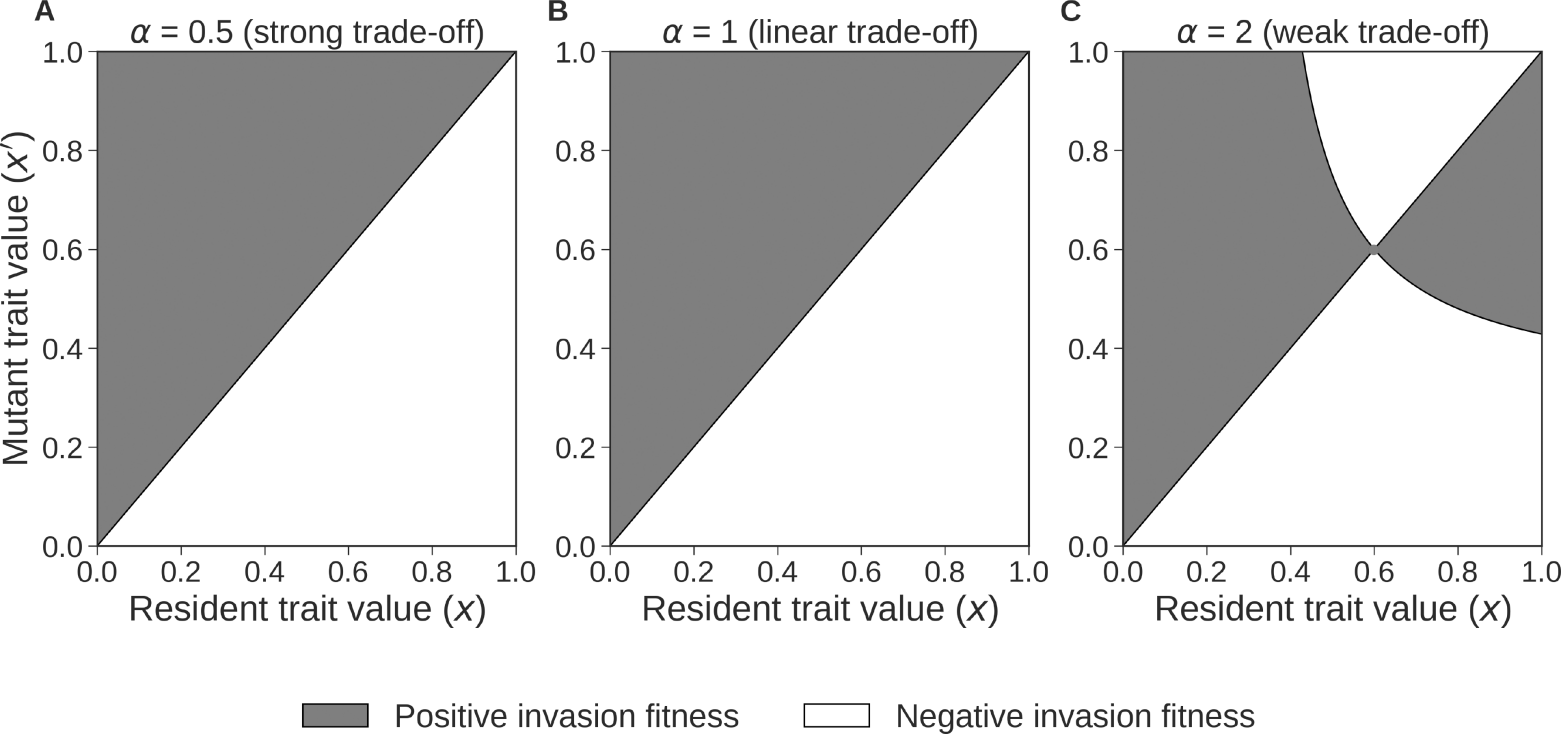
Pairwise invasibility plot (PIP) analyzing evolutionary dynamics through invasion fitness patterns. The trade‐off shape parameter α governs the nonlinearity of the defense–competitiveness trade‐off: α<1 (strong), α=1 (neutral), α>1 (weak). Gray regions (+) indicate successful invasion and white regions (−) indicate failed invasion of a mutant type $x^{\prime }$ in a resident population with trait value x, where x is the prey allocation strategy (ranging from 0 to 1). Invasion fitness zero‐contours separate regions of successful and unsuccessful invasion; the diagonal line ($x=x^{\prime }$) represents resident fitness equilibrium; eco‐evolutionary fixed points, that is, convergence stable strategy (CSS, red dots), occur where zero‐contours intersect the diagonal. Panel A: Strong trade‐off (α<1): selection consistently favors higher competitiveness (increasing x). Panel B: Neutral trade‐off (α=1): selection consistently favors higher competitiveness (increasing x). Panel C: Weak trade‐off (α>1): a single CSS emerges at $\hat{x}=0.599$ for resource level R=2, representing the evolutionarily stable balance between defense and competitiveness.

The eco‐evolutionary results build directly on the ecological outcomes by determining which of the feasible strategies are favored by selection and whether the resulting trait values represent stable evolutionary end points. For conditions of strong and neutral defense–competitiveness trade‐offs, individuals with strategies that have a greater allocation in resource uptake (i.e., higher x) can always invade a population with smaller x (Figure 4A,B). In other words, natural selection would always favor higher x. However, weaker trade‐offs can lead to the emergence of singular evolutionary stable strategies (ESS) (Figure 4C). These singular strategies are also convergent; thus, no other strategy can coexist, for a specific resource supply (Appendix S4). This result is further supported by the geometric analysis of the continuous‐trait case. Under a weak defense–competitiveness trade‐off, impact vectors align with the environmental gradient so that they select a unique singular strategy; because they follow the envelope boundary without intersecting it, they satisfy the geometric condition that rules out coexistence of multiple strategies. This continuous‐trait extension is illustrated in Appendix S5: Figure S1.

A complete derivation of the evolutionary singular target strategy based on the fitness gradient and ZNGI envelope is detailed in Appendix S6. The resulting expression for $\hat{x}$ shows that the magnitude of the singular trait declines with increasing resource level R if and only if α>1. This indicates that as resources become more abundant, prey evolve toward strategies that allocate less to resource acquisition and more to defense against predation. This trend aligns with the results of the ecological approach, whereby higher resource availability under a weak defense–competitiveness trade‐off favored defended prey.

## DISCUSSION

Although growing empirical evidence underscores the ecological significance of intraspecific and interspecific trait variation (Des Roches et al., 2018), key uncertainties persist regarding how variation in prey competitiveness and defenses governs trait prevalence within prey populations, and whether these mechanisms vary predictably across environmental gradients (Guariento et al., 2022). Here, we address this gap by showing how environmental conditions modify the influence of the defense–competitiveness trade‐off on the sorting and evolution of prey traits. By incorporating prey phenotypes specialized in competitiveness or defenses against predators, our model examines how prey traits are sorted and adapt across resource gradients in a diamond food web (Leibold, 1996). Our findings indicate that undefended prey, competition specialists, dominate under conditions of low resource availability, regardless of the shape of the defense–competitiveness trade‐off. When prey traits exhibit a weak trade‐off between defense and competitiveness (i.e., increased defenses incur relatively small reductions in resource acquisition), and prey resources have a high carrying capacity, the resulting high predator densities lead to two key outcomes: either the emergence of a dominant prey type with increased defense or the coexistence of a few prey with high defense levels. These results highlight how the dynamic interaction between bottom‐up and top‐down processes and traits mediates the coexistence of trophically similar prey phenotypes. However, evolutionarily stable coexistence is not possible. In the subsequent discussion, we emphasize the ecological interpretation of these results, their evolutionary implications, and the testable predictions they generate for natural systems.

### Prey trait sorting

Investigating trade‐offs between defense and competitiveness is central to understanding the mechanisms governing coexistence among prey with distinct trait combinations across environmental gradients (Réveillon & Becks, 2024). Our ecological analysis reveals how the defense–competitiveness trade‐off translates into predictable patterns of prey trait sorting along resource gradients. While much previous work has focused on interspecific differences (Chase & Leibold, 2003; Leibold, 1996), our results describe intraspecific trait variation, encompassing the coexistence patterns of multiple genotypes or phenotypes within a single prey population, though the framework applies equally to interspecific competition.

Across all trade‐off shapes, low resource supply consistently favors highly competitive phenotypes (Grover & Holt, 1998). Under resource limitation, prey persistence is determined primarily by minimum resource requirements ($R^{*}$), such that individuals allocating more strongly to resource acquisition achieve positive growth at lower resource levels and exclude alternative phenotypes (Tilman, 1980). In this regime, prey densities remain low, which in turn support lower predator densities, and system dynamics are governed predominantly by bottom‐up control.

As resource availability increases, however, the selective environment shifts. Greater resource supply sustains higher prey biomass, which in turn supports elevated predator densities. This transition increases the strength of top‐down control, creating conditions under which investment in defense may become advantageous (Grover & Holt, 1998; Leibold, 1996). Whether this shift leads to trait diversification or continued competitive dominance depends critically on the curvature of the defense–competitiveness trade‐off. When the trade‐off is neutral to strong, defended phenotypes incur moderate to severe reductions in competitive ability. Under these conditions, investment in defense fails to offset the associated costs in resource acquisition. The resulting fitness landscape is effectively monotonic: Phenotypes maximizing competitiveness achieve the highest net growth rates regardless of predator density (Ehrlich et al., 2017). Importantly, increasing resource availability does not reverse this hierarchy, because even at high resource supply, the marginal fitness cost of additional defense remains greater than the marginal benefit relative to allocating the same effort to resource uptake. Although predator densities rise, the competitively superior phenotype maintains higher growth across the resource gradient because all phenotypes benefit similarly from resource enrichment, while competitive asymmetries persist.

This outcome contrasts with classical keystone predation scenarios, in which predators disproportionately suppress dominant competitors and thereby promote coexistence (Leibold, 1996). Depending on the defense–competitiveness trade‐off relationship, predators act primarily as an additive mortality term rather than an equalizing mechanism. Moreover, trophic feedbacks can amplify competitive asymmetries through apparent competition, but this effect is both trait‐dependent and density‐dependent. The predator population and resulting predation pressure depend on the relative abundances of competitive and defended prey at a given time, so the predation‐mediated fitness reduction is not constant through time and can generate reciprocal feedbacks between prey types under some nutrient conditions (Chesson & Kuang, 2008). Consequently, the system is filtered by competitive ability, favoring the most competitive phenotype across the full resource gradient.

In contrast, weak trade‐offs facilitate ecological coexistence of distinct traits. Here, defended phenotypes have only limited reduction in competitiveness while gaining substantial reductions in predation mortality. This asymmetry generates two viable ecological strategies: Competitive phenotypes dominate under resource limitation when predator densities are low, whereas defended phenotypes gain an advantage in resource‐rich environments characterized by strong top‐down control. This structure supports a stabilizing coexistence mechanism mediated by frequency‐dependent selection. Competitive phenotypes can invade defended populations under low predation pressure, while defended phenotypes can invade competitive populations under high predation pressure. Mutual invasibility under contrasting environmental conditions prevents competitive exclusion and stabilizes coexistence. Importantly, coexistence is restricted to phenotypes with relatively similar trait allocations, indicating weak ecological differentiation and a gradual shift in the optimal strategy along the continuous defense–competitiveness gradient as environmental conditions change (Koffel et al., 2018; Litchman et al., 2007).

These results align with prior theoretical and empirical work linking trait trade‐offs to coexistence and trophic dynamics. For example, variation in growth rates, nutrient uptake traits, or predator susceptibility among phytoplankton strains can generate differential fitness outcomes that shape predator–prey dynamics and community structure (Réveillon & Becks, 2024). Our framework extends these insights by demonstrating how the curvature of defense–competitiveness trade‐offs governs not only which phenotypes persist but also whether ecological coexistence is feasible across environmental gradients.

Taken together, the ecological analysis shows that resource availability mediates the balance between bottom‐up and top‐down controls, while trade‐off shape determines whether this environmental gradient produces competitive exclusion or stabilizing trait coexistence. Understanding how these mechanisms interact is therefore essential for predicting the prevalence and ecological consequences of prey defense strategies in natural systems.

### Prey evolutionary dynamics

This approach analyzes invasion dynamics within the local neighborhood of a resident prey population, focusing on small mutations that can yield evolutionarily stable strategies (ESS). This allows us to distinguish ecological persistence from evolutionary outcomes. We found that the coexistence patterns of different strategies are not robust at the evolutionary scale (Meszéna et al., 2006). This makes evolutionarily stable coexistence unattainable across a continuous spectrum of trait allocation strategies, a pattern also observed in resource allocation strategies for aquatic organisms (Schreiber & Tobiason, 2003). For neutral to strong defense–competitiveness trade‐offs, selection favors higher competitive ability. For weak trade‐offs, we found that as resource supply increases, prey that evolved an allocation strategy toward defensive traits gain an advantage and can successfully dominate the population. These predictions about the selection of defended strategies in high‐resource environments align with a substantial body of research on species interactions. This includes foundational studies by Leibold (1996) and Chase et al. (2000) on plant defenses against herbivory, as well as eco‐evolutionary theoretical frameworks for nutrient–plant–herbivore food webs (Koffel et al., 2018; Loeuille & Loreau, 2004). However, all evolutionarily stable states in such weak trade‐off scenarios act as global CSS, thereby preventing evolutionarily stable coexistence. Prior work in species sorting with few competitors suggests greater coexistence for morphologically similar species (Leibold, 1996), yet our evolutionary‐scale analysis reveals no such trend, possibly because previous investigations were constrained to a more limited trait space.

These findings extend the conclusions of Ehrlich et al. (2017), who highlighted the importance of the trade‐off shape between defenses and competitiveness for trait prevalence. While Ehrlich et al. (2017) established a general framework, showing that convex trade‐offs tend to favor specialists and concave trade‐offs promote generalists or species with intermediate traits, our model details a specific mechanism for a defense–competitiveness trade‐off. The singular strategy ($\hat{x}$) emerging in our system represents an intermediate level of defense investment, and its magnitude and stability are explicitly modulated by both the resource level (*R*) and the trade‐off shape (α). For example, when increased defense entails only limited reductions in competitiveness, the selection for an intermediate strategy $\hat{x}$ is consistent with the predictions of Ehrlich et al. (2017). Furthermore, our study offers a theoretical complement to the empirical observations of Ehrlich et al. (2020), who identified a concave defense‐growth trade‐off in phytoplankton, resulting in community adaptation toward intermediate defense levels in response to grazing pressure. Our work, therefore, elucidates how the interplay between resource availability and the shape of the trade‐off can generate and maintain intermediate defense strategies, providing a mechanistic basis for such patterns observed in natural systems.

Our analysis focused on whether multiple defense–competitiveness strategies can coexist and persist evolutionarily within prey populations. Although trade‐offs could potentially support a wide range of strategies (Becks et al., 2010; Yamamichi & Miner, 2015), our model reveals that evolutionary processes consistently select for a single dominant strategy, preventing stable coexistence. In contrast to our findings, Koffel et al. (2018) conclude that evolutionarily stable coexistence between two distinct primary producer strategies, a highly resistant specialist (i.e., resistant to herbivore grazing) and a fast‐growing tolerant specialist (i.e., tolerant to biomass losses caused by herbivory), is a likely outcome, particularly under high resource supply. This difference might be due to the fact that our model focuses on a single continuous trade‐off axis for defense versus competitiveness, while Koffel et al.'s model involves a three‐way allocation to competition, resistance, and tolerance, where the specific returns on allocation to these separate functions can favor the co‐occurrence of specialized types. Additionally, evolutionarily stable strategies can emerge via evolutionary branching (Geritz et al., 2004). Although our model revealed no evolutionary branching (i.e., adaptive splitting of a single lineage into distinct coexisting phenotypes due to disruptive selection near a singular strategy), other defense–competition frameworks have documented it (e.g., Claessen et al., 2007). This contrast highlights how ecological constraints and the dimensionality of trait allocation can limit diversification, reinforcing the importance of integrating ecological context into evolutionary predictions.

## CONCLUSION

Our study highlights the critical role of resource‐mediated interactions, via trophic interactions, in shaping prey trait prevalence and evolutionary trajectories. By integrating prey strategy sorting and adaptive dynamics through a combined analytical–graphical approach, we show how trait‐level trade‐offs drive diverse competitive outcomes, challenging traditional views on trait optimization and competitive success in diamond‐shaped food webs (Leibold, 1996). These findings emphasize the need to account for multiple regulatory and intrinsic factors when predicting community responses to environmental change (Holt, 2009). Future research should explore several key extensions of this framework. First, investigating how environmental perturbations alter the shape and position of defense–competitiveness trade‐offs would reveal whether these trade‐offs remain static or shift dynamically under changing conditions. Second, examining how such dynamical trade‐offs influence eco‐evolutionary dynamics and population persistence under nonequilibrium conditions could improve predictions of community responses to environmental change. Finally, incorporating trait plasticity and environmental stochasticity into the model would provide a realistic representation of how prey navigate fluctuating resource availability and predation pressure, ultimately improving our ability to predict and manage biodiversity under shifting environmental conditions.

## AUTHOR CONTRIBUTIONS

Rafael Dettogni Guariento wrote the first draft and performed the analysis of the models. Frederico Meirelles‐Pereira, Anderson da Rocha Gripp, Luciana Silva Carneiro, Jayme Magalhães Santangelo, and Adriano Caliman contributed to the review of the manuscript and interpretation of the results.

## CONFLICT OF INTEREST STATEMENT

The authors declare no conflicts of interest.

## Supporting information


Appendix S1.

Appendix S2.

Appendix S3.

Appendix S4.

Appendix S5.

Appendix S6.

## Data Availability

Code (Guariento, 2026) is available in Zenodo at https://doi.org/10.5281/zenodo.21168713.
