# Supplementary material for: Defense–competition trade‐offs shape prey eco‐evolutionary dynamics across environmental gradients
Source: Ecology. 2026 Sep 23;107(9):e70518. doi: 10.1002/ecy.70518 (PMC13601822; doi:10.1002/ecy.70518)
Supplement: Supplementary file 1 [file ECY-107-e70518-s001.pdf]

Appendix S1
Defense–competition trade-offs shape prey eco-evolutionary dynamics across environmental gradients

Rafael Dettogni Guariento
Frederico Meirelles-Pereira
Anderson da Rocha Gripp
Jayme Magalhães Santangelo
Luciana Silva Carneiro
Adriano Caliman

Ecology

Table S1: Model Parameters and State Variables

Table S1 presents the definitions, values, units, and references for all state variables and parameters used in the model. The ecological parameters are based on studies of zooplankton-phytoplankton-fish systems. The carrying capacity values are drawn from fisheries literature and competitive co-efficients are established by ecological theory. The allocation parameters characterizing the defense-competitiveness trade-off are informed by evolutionary optimization principles. To keep dimensional consistency with Eq. (2), resource carrying capacity (K) is varied, and the producer growth rate is computed as $r = \lambda K$ while λ is held constant. Because $\lambda = 1$ mL cells^{-1} day^{-1}, the identity $r = K$ holds numerically, so the absolute densities produced by the model should be interpreted in relative rather than field-realistic terms; the qualitative dynamics and trade-off predictions are independent of this rescaling.

Table S1: Definitions, values, units, and references for the state variables and parameters of the model.

Parameter	Symbol	Value	Units	Reference
State variables				
Resource density	R	–	cells mL^{-1}	–
Prey density	N	–	individuals mL^{-1}	–
Predator density	P	–	individuals L^{-1}	–
Ecological parameters				
Producer growth rate	r	variable ($= \lambda K$)	day^{-1}	Fraisse et al. (2015); Safi et al. (2023)
Prey and predator basal death rate	d_N, d_P	0.3, 0.2	day^{-1}	DeLong & Lyon (2020); Minter et al. (2011)
Maximum predator attack rate on prey	f	4	L individuals^{-1} day^{-1}	DeLong & Lyon (2020); Chan et al. (2017)
Maximum prey consumption rate on resources	g	5	mL individuals^{-1} day^{-1}	Levine et al. (1999); Dubey et al. (2014)
Resource-to-prey conversion factor	e_N	0.1	individuals cells^{-1}	DeLong & Lyon (2020); Li et al. (2015)
Prey-to-predator conversion efficiency	e_P	0.1	–	DeLong & Lyon (2020); Li et al. (2015)
Resource carrying capacity	K	variable	cells mL^{-1}	Ayllón et al. (2012); Campillay-Llanos et al. (2022)
Competition coefficient	λ	1	mL cells^{-1} day^{-1}	Adler et al. (2018); Tilman (1977)
Allocation parameters				
Trait allocation strategy	x	0–1	–	-
Strategy shape parameter	α	variable	–	Farahpour et al. (2018); Jost (2014)

References

[1] Fraisse, S. et al. (2015) Turbulence effects on phytoplankton morphofunctional traits. *Limnology and Oceanography*, 60, 287–301.

[2] Safi, K. et al. (2023) Phytoplankton dynamics, growth and microzooplankton grazing in a subpolar system. *Journal of Marine Systems*, 230, 103783.

[3] DeLong, J.P. and Lyon, S.R. (2020) Temperature alters the shape of predator–prey cycles through effects on underlying mechanisms. *PeerJ*, 8, e9377.

[4] Minter, A., Retkute, R., Brown, G.A. et al. (2011) Prey-dependent mortality rate: a critical parameter in predator–prey models. *Journal of Animal Ecology*, 80, 807–814.

[5] Chan, K., Giron-Nava, A. et al. (2017) Improving the assessment of predator functional responses. *Journal of Animal Ecology*, 86, 701–715.

[6] Levine, S.N., Schindler, D.W. et al. (1999) The impact of zooplankton grazing on phytoplankton in a eutrophic lake. *Limnology and Oceanography*, 44, 1374–1387.

[7] Dubey, B.P., Agrawal, R. and Sharma, S. (2014) Dynamics of Phytoplankton, Zooplankton and Fishery. *Computational and Mathematical Methods in Medicine*, 2014, Article ID 546130.

[8] Li, Y., Schreiber, S.J., Malchow, H. et al. (2015) Restructuring fundamental predator–prey models by recognising prey-dependent conversion efficiency and mortality rates. *Journal of Theoretical Biology*, 385, 1–11.

[9] Ayllón, D., Almodóvar, A., Nicola, G.G. and Elvira, B. (2012) Modelling carrying capacity dynamics for the conservation and management of Atlantic salmon. *Environmental Modelling & Software*, 38, 31–39.

[10] Campillay-Llanos, W., Pérez-Alvarez, M.J. and Momo, F.R. (2022) Coexistence, Energy, and Trophic Cascade in a Three-Species Food Chain Integrating Body Sizes. *Frontiers in Ecology and Evolution*, 10, 821176.

[11] Adler, P.B., Smull, D., Beard, K.H. et al. (2018) Competition and coexistence in plant communities: intraspecific competition is stronger than interspecific competition. *Ecology Letters*, 21, 1319–1329.

[12] Tilman, D. (1977) Resource competition between plankton algae: An experimental and theoretical approach. *Ecology*, 58, 338–348.

[13] Farahpour, F., Saeedghalati, M., Hufton, P. and Lässig, M. (2018) Trade-off shapes diversity in eco-evolutionary dynamics. *eLife*, 7, e36273.

[14] Jost, J. (2014) Optimization and phenotype allocation. *Advances in Applied Mathematics*, 57, 1–17.
