# Supplementary material for: Defense–competition trade‐offs shape prey eco‐evolutionary dynamics across environmental gradients
Source: Ecology. 2026 Sep 23;107(9):e70518. doi: 10.1002/ecy.70518 (PMC13601822; doi:10.1002/ecy.70518)
Supplement: Supplementary file 2 [file ECY-107-e70518-s002.pdf]

Appendix S2
Defense–competition trade-offs shape prey eco-evolutionary dynamics across environmental gradients

Rafael Dettogni Guariento
Frederico Meirelles-Pereira
Anderson da Rocha Gripp
Jayme Magalhães Santangelo
Luciana Silva Carneiro
Adriano Caliman

Ecology

Derivations of Zero Net Growth Isoclines and Supply Points

The supply points (S_R, S_P) depict the resource (i.e., primary producers) and predator densities at equilibrium in the absence of prey, given by $S_R = \frac{r}{\lambda} = K$ and $S_P = 0$ (black dot in Fig. S1). The ZNGI (i.e., equilibrium densities) conducts an invasion analysis by partitioning the environmental (R-P) plane into regions where a prey population j can or cannot persist (Fig. S1). It is derived by setting the net growth (right-hand side of equation 1b) to zero, establishing the requisite relationship between R and P for prey j to persist at equilibrium. A standard ZNGI exhibits a positive net growth below the curve, and negative above it. The ZNGI intersects the R axis at $R^*_j = \frac{d_N}{e_N g_j}$, which signifies the minimum resource level necessary for the species j to survive without predators. In accordance with Tilman's R^* theory, this metric reflects the species' competitive prowess at low resource levels (lower values are advantageous). The slope of the ZNGIs is determined by $\frac{e_N g_j}{f_j}$. Evaluating the ZNGI slope reveals that, to cope with predators, prey must reduce f_j (predator attack rate), which increases the slope and, in turn, enlarges the area below the ZNGI. Crucially, as resource supply increases, predators benefit indirectly through trophic transfer from prey populations. This leads to a significant shift in prey dynamics, from being under bottom-up control to experiencing top-down control, highlighting why defensive strategies might become critical for prey at high resource levels, possibly shaping prey trait prevalence and evolution.

To visualize solutions, the supply point map graphically solves system equilibria by combining impact vectors with ZNGI and supply points. Impact vectors are the collection of a focal population *per capita* effects on the environmental factors, rescaled by the intrinsic turnover rate of that factor (see References in main text). For given environmental conditions, impact vectors map the supply point to equilibrium resource availability and predator density ($\hat{R}$, $\hat{P}$) on the ZNGI (Fig. S1A). As expected, prey presence reduces resource levels through consumption while simultaneously increasing predator abundance by providing food (Leibold 1996). An alternative way to represent these results is to draw the ZNGI and a family of impact vectors directly in the two-dimensional supply point parameter space (or supply point plane). This representation creates a bifurcation diagram, where the region spanned by the impact vectors corresponds to conditions supporting the prey's presence, while the region above the ZNGI corresponds to its absence.

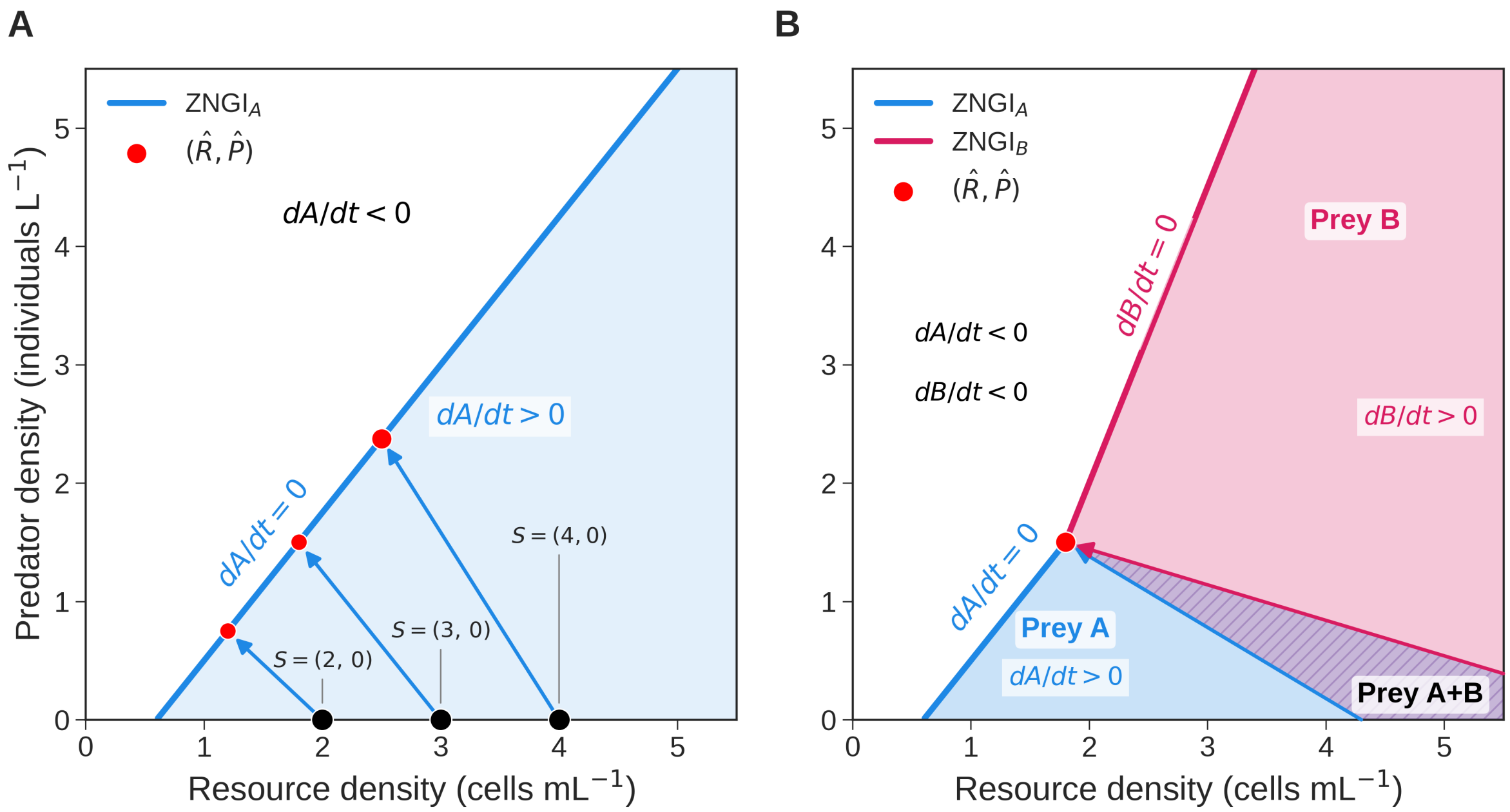

Figure S1: Graphical framework for prey invasion analysis. (A) Single-prey schematic showing the zero net growth isocline (ZNGI; blue), the supply point S (S_R, S_P; black dot), and the impact vector connecting the supply point to the equilibrium point ($\hat{R}, \hat{P}$; red dot) on the isocline. (B) Two-prey schematic showing prey A (blue; higher resource uptake but lower defense) and prey B (magenta; lower resource uptake but higher defense). Colored zones indicate parameter combinations where only A, only B, or both prey persist; the coexistence zone is bounded by the two impact vectors that emerge from the ZNGI intersection. In the region above both isoclines, neither prey persists.

Numerical confirmation of strategy replacement along the resource gradient

Figure S2 provides the numerical complement to the graphical invasion analysis shown in Figure S1 and used in the main text. Panel A summarizes the weak trade-off case (i.e., $\alpha > 1$) in the supply-point plane, where each black dot corresponds to one resource supply level ($S_R = K$) with $S_P = 0$. Panels B–H then show the stable ecological equilibrium reached for each selected supply point, making explicit which prey strategies persist as environmental conditions change.

Read from Panel B to Panel H, the sequence shows how community composition reorganizes along enrichment. At low resource supply, equilibria are dominated by more competitive and less-defended prey strategies. As resource supply increases, predator abundance rises through trophic transfer, strengthening top-down control and progressively favoring prey with greater defense. Therefore, Figure S2 is the numerical evidence supporting the main-text result that enrichment can shift persistence from competitive to more defended prey strategies under weak defense-competitiveness trade-offs.

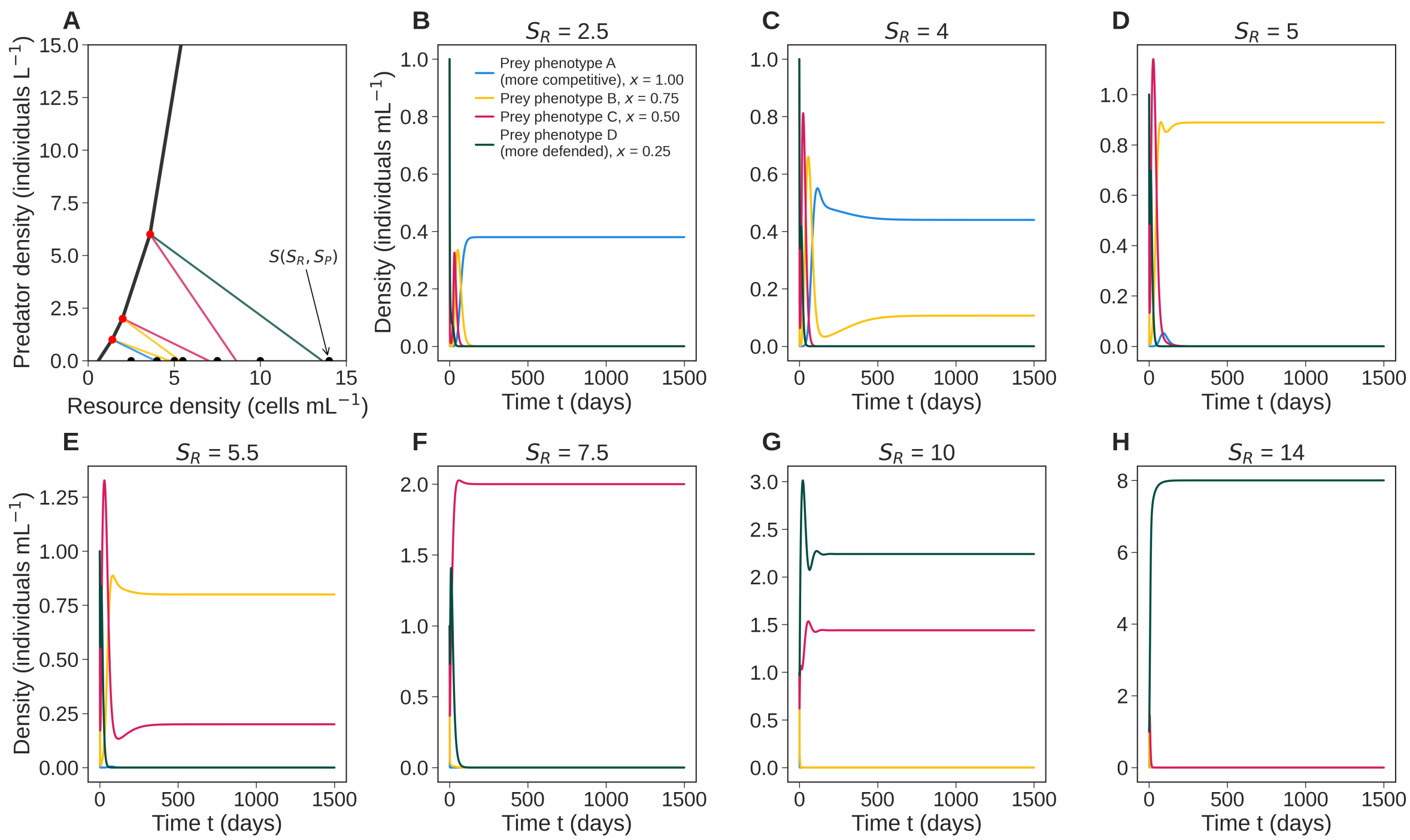

Figure S2: Numerical confirmation of prey strategy sorting along the resource gradient under weak defense-competitiveness trade-offs. **Panel A:** Resulting Zero Net Growth Isocline (ZNGI) diagram from the outermost ZNGIs of four prey populations, with seven selected supply points ((S_R, S_P); black dots) along the $S_P = 0$ cross-section and increasing S_R. These points span distinct community-composition regions defined by ZNGIs and impact vectors. **Panels B-H:** Equilibrium prey densities (N_i) for each of the four prey populations at the seven supply points shown in Panel A. Together, the panels show that increasing resource supply (i.e., increasing $K = r/\lambda$) changes which strategy persists, with equilibria shifting from more competitive prey at low supply to more defended prey at high supply.
