# Supplementary material for: Defense–competition trade‐offs shape prey eco‐evolutionary dynamics across environmental gradients
Source: Ecology. 2026 Sep 23;107(9):e70518. doi: 10.1002/ecy.70518 (PMC13601822; doi:10.1002/ecy.70518)
Supplement: Supplementary file 3 [file ECY-107-e70518-s003.pdf]

Appendix S3
Defense–competition trade-offs shape prey eco-evolutionary dynamics across environmental gradients

Rafael Dettogni Guariento
Frederico Meirelles-Pereira
Anderson da Rocha Gripp
Jayme Magalhães Santangelo
Luciana Silva Carneiro
Adriano Caliman

Ecology

Ecological Equilibria and Stability Analysis

This supplement details the derivation of ecological equilibria and local stability conditions for the diamond food-web model presented in the main text. We begin with the single-prey case and then extend the analysis to two competing prey populations. Equilibrium densities are denoted by $\hat{R}$, $\hat{N}$, and $\hat{P}$ throughout.

Part I: Single Prey Population

We first consider one prey population with a fixed anti-predator strategy. In this setting, two equilibrium regimes are possible: prey extinction and prey persistence.

I.1 Equilibrium Conditions

Trivial Equilibrium (E0). When prey is absent ($\hat{N}_A = 0$), equation (1a) yields $\hat{R} = K = S_R$. Equation (1c) then implies that predators cannot persist, so $\hat{P} = 0 = S_P$. This corresponds to the prey-free supply point.

Predator-free Equilibrium ($\mathbf{E_R}$). When prey persists but predators are absent ($\hat{N}_A > 0$, $\hat{P} = 0$), equation (1b) gives $\hat{R} = R^*_A = \frac{d_N}{e_N g_A}$. Substituting into (1a) yields

$$\hat{N}_A = \frac{\lambda(K - \hat{R})}{g_A}. \tag{S1}$$

This equilibrium is feasible when $\hat{R} < K$. It is resistant to predator invasion when $e_P f_A \hat{N}_A - d_P < 0$.

Non-trivial Equilibrium (EA). When prey persists ($\hat{N}_A > 0$), equation (1b) at equilibrium requires:

$$e_N g_A \hat{R} - f_A \hat{P} - d_N = 0$$

This relation defines prey A's zero net growth isocline, ZNGI$_A$, so the equilibrium pair ($\hat{R}$, $\hat{P}$) must lie on this line.

A second equilibrium condition follows by eliminating $\hat{N}_A$ between equations (1b) and (1c):

$$S_P - \hat{P} = \beta(\hat{R}, \hat{P})(S_R - \hat{R}) \tag{S2}$$

where β captures the prey's relative effect on the two regulating factors:

$$\beta(\hat{R}, \hat{P}) = -\frac{e_N \lambda f_A \hat{P}}{d_P e_P g_A} \tag{S3}$$

Because all parameters are positive, $\beta < 0$. Geometrically, $(\hat{R},\ \hat{P})$ is given by the intersection of ZNGI$_A$ and the impact ray defined by equation (S2). Once this pair is known, $\hat{N}_A$ follows from equation (1b) or (1c):

$$\hat{N}_A = \frac{\lambda(S_R - \hat{R})}{g_A} \tag{S4}$$

This equilibrium is biologically feasible only when $S_R > \hat{R}$, meaning the supply point lies below ZNGI$_A$.

I.2 Stability Analysis

The Jacobian matrix of the single-prey system, with state variables ordered as (N_A, R, P), is:

$$J(N_A, R, P) = \begin{bmatrix} e_N g_A R - f_A P - d_N & e_N g_A N_A & -f_A N_A \\ -g_A R & \lambda(K - 2R) - g_A N_A & 0 \\ e_P f_A P & 0 & e_P f_A N_A - d_P \end{bmatrix} \tag{S5}$$

Stability of E0. At $(0, K, 0)$:

$$J(0, K, 0) = \begin{bmatrix} e_N g_A K - d_N & 0 & 0 \\ -g_A K & -\lambda K & 0 \\ 0 & 0 & -d_P \end{bmatrix} \tag{S6}$$

Since the matrix is triangular, its eigenvalues are the diagonal entries. Therefore, local stability (all eigenvalues negative) reduces to:

$$e_N g_A K - d_N < 0 \tag{S7}$$

Geometrically, E0 is stable precisely when $(K, 0)$ lies above ZNGI$_A$, so prey A has negative invasion growth.

Stability of EA. At $(\hat{N}_A, \hat{R}, \hat{P})$:

$$J(\hat{N}_A, \hat{R}, \hat{P}) = \begin{bmatrix} 0 & e_N g_A \hat{N}_A & -f_A \hat{N}_A \\ -g_A \hat{R} & \lambda(K - 2\hat{R}) - g_A \hat{N}_A & 0 \\ e_P f_A \hat{P} & 0 & e_P f_A \hat{N}_A - d_P \end{bmatrix} \tag{S8}$$

The (1,1) term is zero because $\hat{N}_A > 0$ implies that the ZNGI condition is satisfied at equilibrium. If $\hat{P} > 0$, equation (1c) gives $e_P f_A \hat{N}_A = d_P$, so the (3,3) term is also zero. Local stability depends on parameter values and can be assessed via the characteristic polynomial. For a cubic $\lambda^3 + a_1\lambda^2 + a_2\lambda + a_3$, the Routh-Hurwitz conditions are $a_1 > 0$, $a_3 > 0$, and $a_1 a_2 > a_3$.

Summary: In the one-prey system, E0 is stable when prey cannot invade, while $\mathrm{E_R}$ or EA require additional stability checks beyond feasibility.

Part II: Two Competing Prey Populations

We now extend the same framework to two prey populations (A and B) with distinct trait values, yielding four equilibrium types.

II.1 Equilibrium Conditions

E0: Both prey absent $(N_A = N_B = 0)$. As in Part I, this gives $\hat{R} = K = S_R$ and $\hat{P} = 0 = S_P$.

EA: Only prey A present $(N_A > 0,\ N_B = 0)$. Here, the equilibrium pair $(\hat{R},\ \hat{P})$ lies on ZNGI$_A$:

$$e_N g_A \hat{R} - f_A \hat{P} - d_N = 0$$

with $\hat{N}_A$ obtained as in Part I. Feasibility again requires the supply point to lie below ZNGI$_A$.

EB: Only prey B present $(N_A = 0,\ N_B > 0)$. This case is symmetric to EA after swapping indices.

EAB: Coexistence $(N_A > 0,\ N_B > 0)$. Both ZNGI conditions must hold simultaneously:

$$e_N g_i \hat{R} - f_i \hat{P} - d_N = 0 \quad \text{for } i = A, B$$

Thus, $(\hat{R},\ \hat{P})$ is located at the intersection of ZNGI$_A$ and ZNGI$_B$. The corresponding $\hat{N}_A$ and $\hat{N}_B$ are obtained from the linear system defined by equations (1b) and (1c) at equilibrium; this solution is unique when impact vectors are non-collinear.

Coexistence is feasible ($\hat{N}_A > 0$ and $\hat{N}_B > 0$) only when the supply point lies within the cone spanned by the two prey impact vectors and originating at the ZNGI intersection (Leibold, 1996).

II.2 Stability Analysis

The Jacobian matrix for the two-prey system, with state variables ordered as (N_A, N_B, R, P), is:

$$J = \begin{bmatrix} e_N g_A R - f_A P - d_N & 0 & e_N g_A N_A & -f_A N_A \\ 0 & e_N g_B R - f_B P - d_N & e_N g_B N_B & -f_B N_B \\ -g_A R & -g_B R & \lambda(K - 2R) - g_A N_A - g_B N_B & 0 \\ e_P f_A P & e_P f_B P & 0 & e_P f_A N_A + e_P f_B N_B - d_P \end{bmatrix} \tag{S9}$$

Stability of E0. At $(0, 0, K, 0)$, the Jacobian is block diagonal. Stability requires both prey to have negative invasion fitness:

$$\begin{aligned} e_N g_A K - d_N &< 0 \\ e_N g_B K - d_N &< 0 \end{aligned} \tag{S10}$$

Geometrically, E0 is stable exactly when the supply point lies above both ZNGIs.

Stability of EA. At $(\hat{N}_A, 0, \hat{R}, \hat{P})$, the Jacobian has a block structure. Expanding along the second row shows that the spectrum contains the single-prey eigenvalues together with:

$$\lambda_B = e_N g_B \hat{R} - f_B \hat{P} - d_N \tag{S11}$$

which is prey B's invasion fitness. Hence, EA is stable exactly when $(\hat{R},\ \hat{P})$ lies above ZNGI$_B$, so prey B cannot invade.

Stability of EB. Symmetric to EA after exchanging A and B.

Stability of EAB. At coexistence, both prey diagonal entries are zero because the ZNGI conditions are satisfied. Local stability is not determined by $\det J > 0$ alone; it depends on the full characteristic polynomial (e.g., a 4th order Routh-Hurwitz test). In the graphical framework, stable coexistence corresponds to mutual invasibility: each prey can invade the other's single-prey equilibrium, which occurs when the ZNGIs cross and each impact vector points into the other's persistence region (the coexistence cone).

Geometrically, this condition links stability to the relative orientation of the ZNGIs at their intersection and to the alignment of the corresponding impact vectors (Koffel *et al.*, 2018; Leibold, 1996; Meszéna *et al.*, 2006). Coexistence is stable when the prey type with the steeper ZNGI also has the steeper impact vector, which generates negative frequency dependence.

References

Koffel, T., Daufresne, T., Massol, F. & Klausmeier, C.A. (2018) Plant Strategies along Resource Gradients. *The American Naturalist*, **192**, 360–378.

Leibold, M.A. (1996) A Graphical Model of Keystone Predators in Food Webs: Trophic Regulation of Abundance, Incidence, and Diversity Patterns in Communities. *The American Naturalist*, **147**, 784–812.

Meszéna, G., Gyllenberg, M., Pásztor, L. & Metz, J.A. (2006) Competitive exclusion and limiting similarity: A unified theory. *Theoretical Population Biology*, **69**, 68–87.
