# Supplementary material for: Defense–competition trade‐offs shape prey eco‐evolutionary dynamics across environmental gradients
Source: Ecology. 2026 Sep 23;107(9):e70518. doi: 10.1002/ecy.70518 (PMC13601822; doi:10.1002/ecy.70518)
Supplement: Supplementary file 4 [file ECY-107-e70518-s004.pdf]

Appendix S5
Defense–competition trade-offs shape prey eco-evolutionary dynamics across environmental gradients

Rafael Dettogni Guariento
Frederico Meirelles-Pereira
Anderson da Rocha Gripp
Jayme Magalhães Santangelo
Luciana Silva Carneiro
Adriano Caliman

Ecology

Continuous Strategy Set and Global Envelope

By superimposing a large number of ZNGIs sampled from the trait space x, it is possible to identify a discrete envelope. When the number of ZNGIs sampled tends toward infinity, this discrete envelope tends toward a smooth curve called the outer geometrical envelope of the ZNGI family. The representation of the geometrical envelope, which is the boundary of a family of ZNGIs, provides a natural way to examine prey phenotypes sorting along environmental gradients. The concept of a geometric envelope is helpful in understanding the dynamics of prey phenotype and allows an extension of invasion analysis to a continuum of competitors.

The global envelope depicted in Figure S1 is the outer boundary of the family of ZNGIs generated by the continuous trait space $0 \leq x \leq 1$, computed here for the weak trade-off case ($\alpha = 2$). Every point on this envelope lies on a ZNGI that is tangent to the envelope, meaning the strategy x at that point simultaneously satisfies two conditions: zero net growth [Eq. (S1)] and vanishing fitness gradient [Eq. (S2)]:

$$w(x, E) = e_N g x R - f x^2 P - d_N = 0 \tag{S1}$$

$$\frac{\partial w}{\partial x}(R, P, x) = 0 \tag{S2}$$

Solving this system eliminates x and yields the envelope as an explicit curve $\hat{P}(R)$ in the resource–predator plane.

Step 1. Solve Eq. (S1) for the equilibrium predator density as a function of x:

$$\hat{P} = \frac{e_N g R x - d_N}{f x^2} \tag{S3}$$

Step 2. Apply the envelope condition Eq. (S2). Differentiating w with respect to x and setting to zero:

$$e_N g R - 2 P f x = 0 \tag{S4}$$

Solving for the singular strategy $\hat{x}$:

$$\hat{x} = \frac{e_N g R}{2 P f} \tag{S5}$$

Step 3. Substitute Eq. (S5) into Eq. (S3) and solve for P to obtain the envelope curve in the (R, P) plane:

$$\hat{P} = \frac{e_N^2 g^2 R^2}{4 d_N f} \tag{S6}$$

Equation (S6) is the envelope curve plotted in Figure S1. It is a parabola in resource level R: as resource availability increases, the envelope rises quadratically, encompassing an expanding region of the (R, P) plane within which some prey strategy can achieve zero net growth. No individual ZNGI can lie above this envelope, confirming it as the global outer boundary of the ZNGI family.

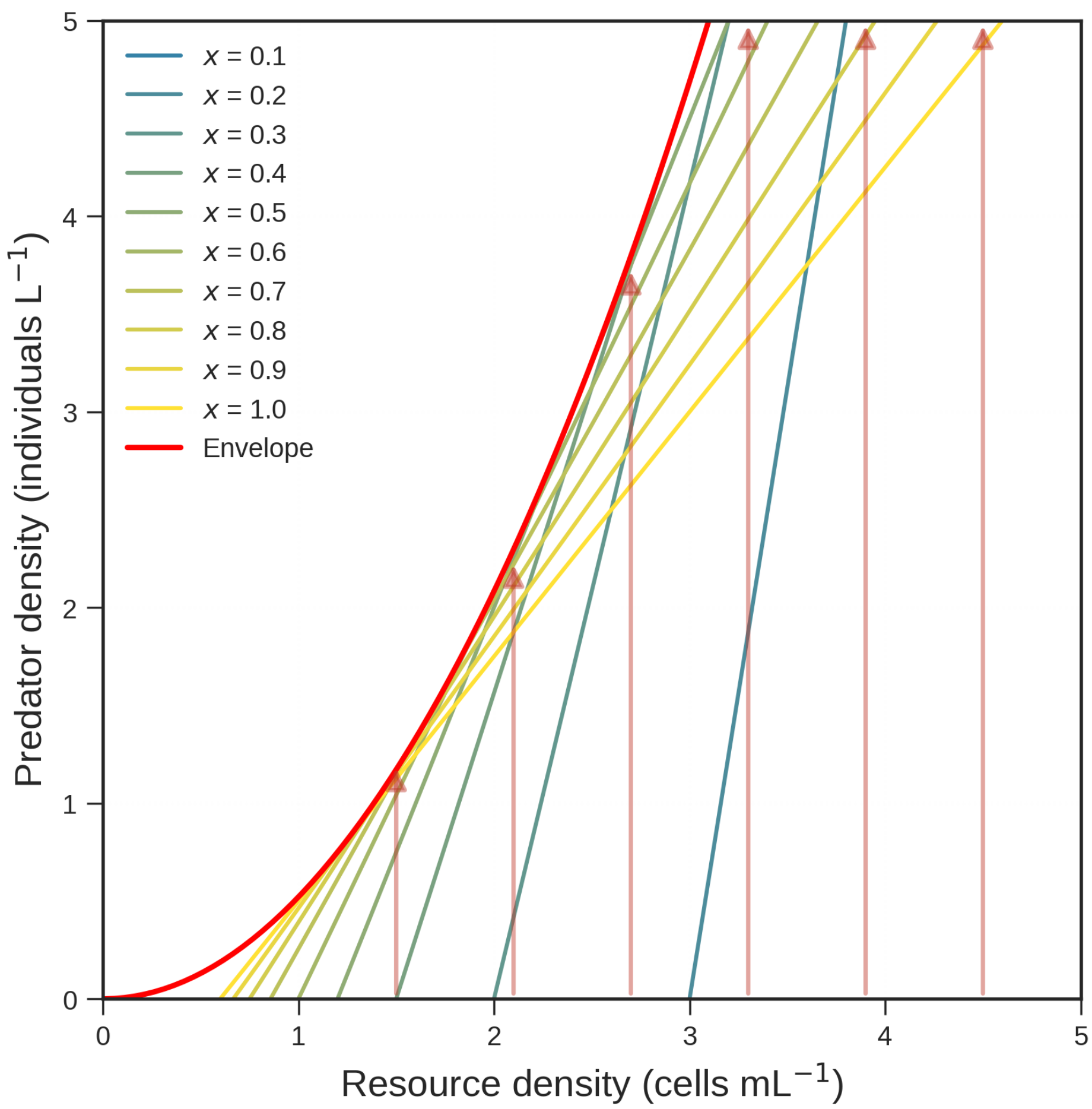

Figure S1: Graphical representation of a continuous strategy set for prey populations competing for resources (R) and under predation (P) for the weak defense-competitiveness trade-off case ($\alpha > 1$). The global envelope (thick red curve) encompasses the Zero Net Growth Isoclines (ZNGIs) for the continuous trait space where $0 \leq x \leq 1$. A representative subset of individual ZNGIs (colored lines) illustrates specific strategies, with their corresponding strategy (x). The impact vectors are represented as dashed red lines. The envelope's global nature is demonstrated by all individual ZNGIs lying below it, indicating that no strategy can achieve positive growth in the region above the envelope. The distinct prey populations represent different allocation strategies (x) for base values of $g_i = 0.6$, $f_i = 0.5$.
