# Supplementary material for: Defense–competition trade‐offs shape prey eco‐evolutionary dynamics across environmental gradients
Source: Ecology. 2026 Sep 23;107(9):e70518. doi: 10.1002/ecy.70518 (PMC13601822; doi:10.1002/ecy.70518)
Supplement: Supplementary file 5 [file ECY-107-e70518-s005.pdf]

# Appendix S6
# Defense–competition trade-offs shape prey eco-evolutionary dynamics across environmental gradients

Rafael Dettogni Guariento
Frederico Meirelles-Pereira
Anderson da Rocha Gripp
Jayme Magalhães Santangelo
Luciana Silva Carneiro
Adriano Caliman

*Ecology*

## Derivation of the Evolutionarily Singular Strategy $\hat{x}$

This supplement derives the closed-form expression for the evolutionarily singular trait allocation strategy $\hat{x}$, the prey strategy at which the selection gradient vanishes and natural selection has no directional preference. Ecologically, $\hat{x}$ represents the equilibrium balance between resource acquisition (competitiveness) and predator avoidance (defense) that a prey population will evolve toward under the adaptive dynamics framework.

The derivation proceeds from the geometrical envelope of the Zero Net Growth Isoclines (ZNGIs). Every point on this envelope corresponds to a ZNGI that is tangent to the envelope, meaning that the strategy $x$ generating that ZNGI satisfies two simultaneous conditions [Eqs. (S1)–(S2) below, corresponding to Eqs. (S1)–(S2) in Appendix S5]:

$$w(x, E) = e_N gxR - fx^{\alpha}P - d_N = 0 \tag{S1}$$

$$\frac{\partial w}{\partial x}(R, P, x) = 0 \tag{S2}$$

Equation (S1) states that the focal prey strategy is at zero net growth (i.e., it lies on its own ZNGI), while Equation (S2) states that no neighboring strategy can achieve higher fitness — the hallmark of a singular point on the trait continuum. Solving this system jointly yields $\hat{x}$ as a function of environmental conditions $R$ and $P$.

**Step 1.** Isolate the equilibrium predator density $\hat{P}$ from Eq. (S1):

$$\hat{P} = \frac{e_N gRx - d_N}{fx^{\alpha}} \tag{S3}$$

**Step 2.** Apply the envelope condition Eq. (S2) — differentiating $w$ with respect to $x$ and setting to zero:

$$-P\alpha fx^{\alpha-1} + e_N gR = 0 \tag{S4}$$

Rearranging to express $x$ in terms of $P$ and $R$:

$$x = \left(\frac{P\alpha f}{Re_N g}\right)^{\frac{-1}{\alpha-1}} \tag{S5}$$

**Step 3.** Substitute Eq. (S3) into Eq. (S5) to eliminate $P$ and obtain the singular strategy as a function of $R$ alone:

$$\hat{x} = \frac{\alpha d_N}{e_N g R(\alpha - 1)} \tag{S6}$$

Equation (S6) is the evolutionarily singular trait value. Inspection of this expression reveals two key ecological predictions. First, $\hat{x}$ is a decreasing function of $R$ when $\alpha > 1$ (weak trade-off): as resource availability increases, prey evolve toward lower $x$, meaning they allocate less to resource acquisition and more to defense. This result is consistent with the ecological analysis (main text), where richer environments with higher predator pressure favor more defended prey. Second, when $\alpha \leq 1$ (neutral or strong trade-offs), $\hat{x}$ is either undefined or negative, confirming that no interior singular strategy exists and selection consistently favors the maximum competitive allocation ($x \to 1$).
