# Supplementary material for: Defense–competition trade‐offs shape prey eco‐evolutionary dynamics across environmental gradients
Source: Ecology. 2026 Sep 23;107(9):e70518. doi: 10.1002/ecy.70518 (PMC13601822; doi:10.1002/ecy.70518)
Supplement: Supplementary file 6 [file ECY-107-e70518-s006.pdf]

# Appendix S4
# Defense–competition trade-offs shape prey eco-evolutionary dynamics across environmental gradients

Rafael Dettogni Guariento
Frederico Meirelles-Pereira
Anderson da Rocha Gripp
Jayme Magalhães Santangelo
Luciana Silva Carneiro
Adriano Caliman

*Ecology*

## Pairwise Invasibility Plots - Equilibria and Stability

Prey evolutionary dynamics can also be studied graphically (Geritz et al., 1998; Brännström et al., 2013). The invasion fitness characterizes the fitness landscape from the perspective of a rare mutant. In a large (effectively infinite) population, only mutants with trait values $x'$ for which its fitness within the resident population $x$ is positive are able to successfully invade. The usual outcome of a successful invasion is that the mutant replaces the resident, and the fitness landscape as experienced by a rare mutant changes. Pairwise invasibility plots (PIPs) are often used to study the consequences of a series of invasions. These plots show, for each resident trait value $x$, all mutant trait values $x'$ for which its fitness is positive. Figure S1 shows PIPs corresponding to different scenarios. The gray area corresponds to pairs $x$ and $x'$ for which a mutant with trait value $x'$ can successfully invade a resident population with trait value $x$, i.e., for which the fitness of the mutant traits $> 0$. Note that fitness of the mutant trait is zero along the diagonal $x' = x$. In these PIPs, the fitness landscapes as experienced by a rare mutant correspond to vertical lines along which the resident trait value $x$ is constant (Koffel et al., 2016).

The evolutionarily singular strategies in a PIP are found where the boundary of the region of positive invasion fitness intersects the diagonal. The singular strategy in Figure S1A is a fitness maximum, i.e., an evolutionarily stable strategy, since the invasion fitness is negative both above and below the singular strategy. It is also a convergence stable strategy since nearby monomorphic populations can be invaded by mutants closer to the strategy (i.e., it is an attractor of monomorphic evolutionary dynamics with small mutational steps). Figure S1 shows that all four logical combinations of evolutionary stability and convergence stability can be realized. The singular strategies in the two PIPs in the first row are evolutionarily stable while those in the second column are convergence stable. A singular strategy that is both evolutionarily stable and convergence stable is a possible endpoint of evolutionary change, while a convergence stable strategy that is a fitness minimum is an evolutionary branching point at which the population eventually becomes dimorphic (Geritz et al., 1997).

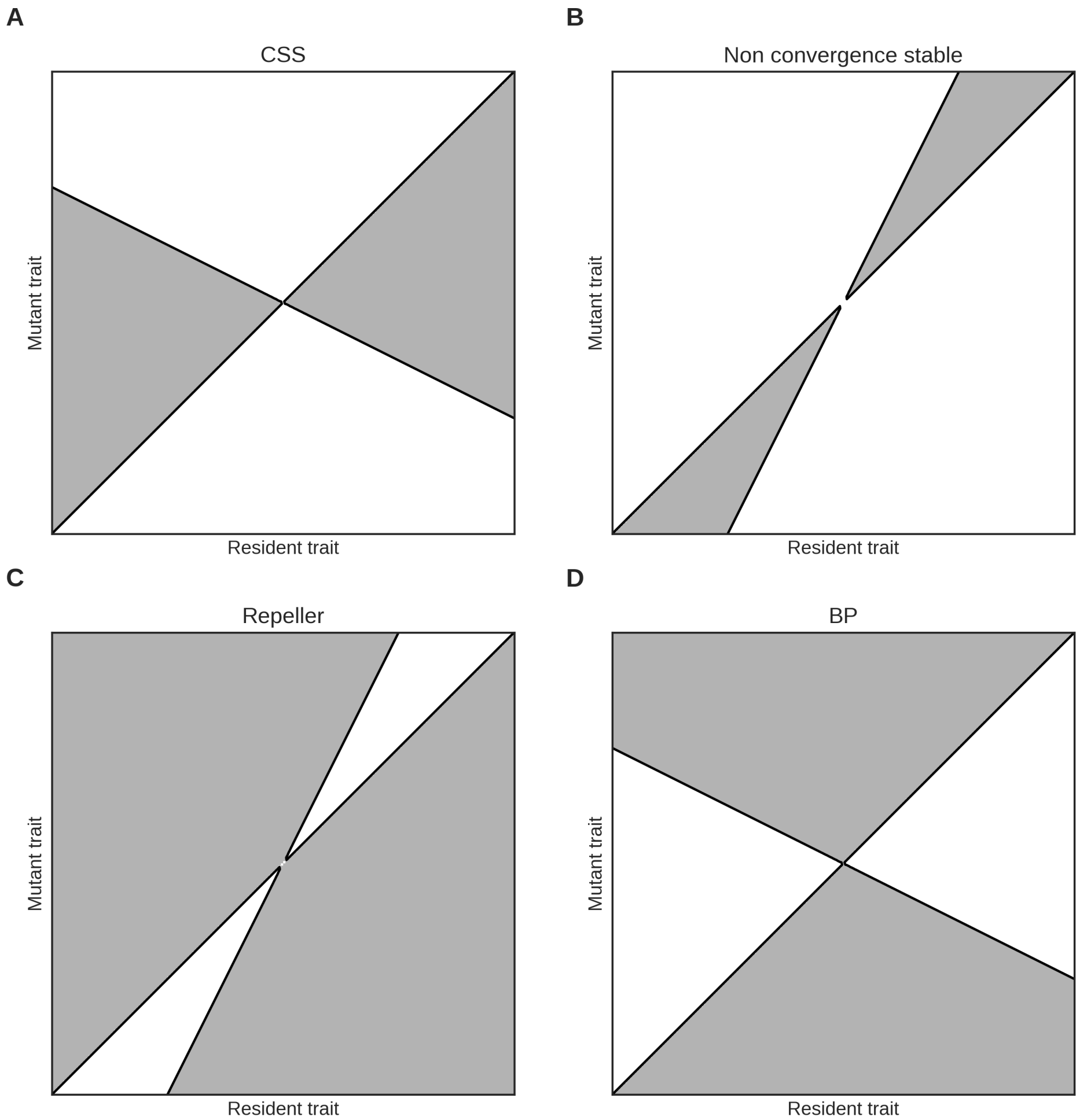


Figure S1: Four logical combinations of evolutionary stability and convergence stability for a singular strategy represented in pairwise invasibility plots (PIPs). The gray area represent positive invasion fitness for the mutant strategy, the white area represent negative invasion fitness. (A) Evolutionarily stable and convergence stable. A possible endpoint of evolution: the strategy can be attained gradually and then it will resist any invaders successfully. (B) Evolutionarily stable but not convergence stable. Such singular strategies should rarely be realized in nature: although the strategy cannot be invaded once it is realized, evolution starting from any nearby strategy will gradually lead away from the singular strategy. (C) Convergence stable but not evolutionarily stable. A scenario where a population can become dimorphic: the singular strategy can be established gradually, but then it can be invaded by mutants both above and below the resident strategy at the same time. (D) Neither evolutionarily stable nor convergence stable. As in (b), a monomorphic population will evolve away from the singular strategy. It is still possible that a dimorphic population will arise, if coexistence is supported, but it is likely to happen through a large mutational step rather than the gradual process of evolutionary branching.

# References


Geritz, S.A.H., Kisdi, É., Meszéna, G. and Metz, J.A.J. (1997) Dynamics of adaptation and evolutionary branching. *Physical Review Letters*, 78, 2024–2027.

Geritz, S.A.H., Kisdi, É., Meszéna, G. and Metz, J.A.J. (1998) Evolutionarily singular strategies and the adaptive growth and branching of the evolutionary tree. *Evolutionary Ecology*, 12, 35–57.

Brännström, Å., Johansson, J. and von Festenberg, N. (2013) The hitchhiker's guide to adaptive dynamics. *Games*, 4, 304–328.

Koffel, T., Daufresne, T. and Massol, F. (2016) Geometrical envelopes: extending graphical contemporary niche theory to communities and eco-evolutionary dynamics. *Journal of Theoretical Biology*, 407, 271–289.
